# βIII-Tubulin: A novel mediator of chemoresistance and metastases in pancreatic cancer

**DOI:** 10.18632/oncotarget.2946

**Published:** 2014-12-10

**Authors:** Joshua A. McCarroll, George Sharbeen, Jie Liu, Janet Youkhana, David Goldstein, Nigel McCarthy, Lydia F. Limbri, Dominic Dischl, Güralp O. Ceyhan, Mert Erkan, Amber L. Johns, Andrew V. Biankin, Maria Kavallaris, Phoebe A. Phillips

**Affiliations:** ^1^ Pancreatic Cancer Translational Research Group, Lowy Cancer Research Centre, Prince of Wales Clinical School, University of New South Wales (UNSW Australia), Sydney, Australia; ^2^ Children's Cancer Institute, Lowy Cancer Research Centre, UNSW Australia, Sydney, Australia; ^3^ ARC Centre of Excellence in Convergent Bio-Nano Science and Technology, Australian Centre for NanoMedicine, UNSW, Australia; ^4^ Prince of Wales Hospital, Prince of Wales Clinical School, Sydney, NSW, Australia; ^5^ Department of Surgery, Klinikum Rechts der Isar, Technische Universität München, Munich, Germany; ^6^ Department of Surgery Koc University School of Medicine, Istanbul, Turkey; ^7^ The Kinghorn Cancer Centre, Cancer Program, Garvan Institute of Medical Research, Darlinghurst, Sydney, Australia; ^8^ Wolfson Wohl Cancer Research Centre, Institute of Cancer Sciences, University of Glasgow, Bearsden, Glasgow, Scotland G61 1BD, United Kingdom

**Keywords:** Pancreatic cancer, chemoresistance, tumor growth, metastases, βIII-tubulin

## Abstract

Pancreatic cancer is a leading cause of cancer-related deaths in Western societies. This poor prognosis is due to chemotherapeutic drug resistance and metastatic spread. Evidence suggests that microtubule proteins namely, β-tubulins are dysregulated in tumor cells and are involved in regulating chemosensitivity. However, the role of β-tubulins in pancreatic cancer are unknown. We measured the expression of different β-tubulin isotypes in pancreatic adenocarcinoma tissue and pancreatic cancer cells. Next, we used RNAi to silence βIII-tubulin expression in pancreatic cancer cells, and measured cell growth in the absence and presence of chemotherapeutic drugs. Finally, we assessed the role of βIII-tubulin in regulating tumor growth and metastases using an orthotopic pancreatic cancer mouse model. We found that βIII-tubulin is highly expressed in pancreatic adenocarcinoma tissue and pancreatic cancer cells. Further, we demonstrated that silencing βIII-tubulin expression reduced pancreatic cancer cell growth and tumorigenic potential in the absence and presence of chemotherapeutic drugs. Finally, we demonstrated that suppression of βIII-tubulin reduced tumor growth and metastases in vivo. Our novel data demonstrate that βIII-tubulin is a key player in promoting pancreatic cancer growth and survival, and silencing its expression may be a potential therapeutic strategy to increase the long-term survival of pancreatic cancer patients.

## INTRODUCTION

Pancreatic ductal adenocarcinoma (PDA) is a devastating disease that ranks as the fourth leading cause of cancer-related death in Western societies, with a 5-year survival rate of 6-7% [1-5]. This poor prognosis is due to PDA's propensity to acquire resistance to chemotherapeutic agents and metastasize [8, 9]. The result is that our current chemotherapeutic treatments only extend patient survival by ~8–16 weeks [10]. These statistics highlight the imperative to identify effective therapeutic targets for this devastating disease.

The tubulin/microtubule network is recognized as a key player in cancer chemoresistance [11]. Microtubules are tube-like assemblies of α-and β-tubulin heterodimers, that form part of the cell cytoskeleton and play critical roles in regulating mitosis and intracellular transport [11]. β-tubulin has seven different isotypes (βI, βII, βIII, βIVa, βIVb, βV, βVI) that exhibit distinct tissue expression profiles [11]. Notably, all of the β-tubulin isotypes share a high degree of homology and are distinguished by their unique carboxy terminal tail which is subject to post-translational modifications [11]. The importance of microtubules as therapeutic targets for cancer is highlighted by the clinical use of tubulin binding agents (TBAs) which target β-tubulin. At high concentrations these agents induce mitotic arrest and cause cell death [12]. However, therapeutic applications of TBAs are often marred by resistance, which is often correlated to differential expression of specific β-tubulin isotypes [11]. Clinical studies have reported high expression of βIII-tubulin in several cancers including lung, breast, prostate, gastric and melanoma [13-18]. Under non-pathological conditions βIII-tubulin expression is primarily restricted to neurons, and sertoli cells in the testis, and at low levels in other tissues [19, 20]. Interestingly, its upregulation in cancer cells has been correlated to decreased progression-free or overall survival and resistance to chemotherapeutic agents [21]. Functional studies have confirmed the importance of βIII-tubulin in regulating sensitivity to chemotherapeutic agents in non-small cell lung cancer (NSCLC), ovarian cancer and prostate cancer cells [22-25].

Up-regulation of βIII-tubulin has been observed in advanced PDA patient tissue specimens and cell lines [26]. Interestingly, aberrant βIII-tubulin expression in PDA cells was associated with activation of kRAS (oncogene commonly associated with PDA) and appeared to be progressively upregulated in pancreatic intraepithelial neoplasias (PanIN) 1 to 3, the precursor lesions of PDA [26]. Despite this strong correlation, no functional role for βIII-tubulin has been established in pancreatic cancer. Using a gene-silencing approach we silenced βIII-tubulin expression in pancreatic cancer cells and determined its role in regulating chemosensitivity, cell growth, tumorigenesis and metastases. We report for the first time that silencing βIII-tubulin in pancreatic cancer cells *in vitro* 1) decreases clonogenicity; 2) decreases anchorage-dependent and independent proliferation; 3) increases apoptosis and anoikis; and 4) increases sensitivity to chemotherapy drugs including gemcitabine and the TBAs paclitaxel and vincristine. Notably, we demonstrate the importance of βIII-tubulin in regulating tumor growth and metastases in a clinically-relevant orthotopic pancreatic cancer mouse model.

## RESULTS

### βIII-tubulin is expressed in human pancreatic tumor cells

**Figure 1:**
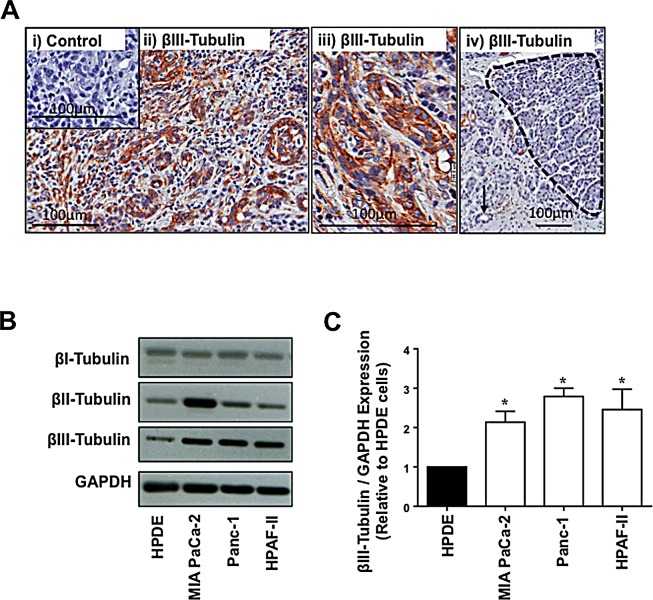
βIII-tubulin expression in PDA patient tissue and PDA cell lines A) Immunohistochemistry for βIII-tubulin in a representative human PDA tissue specimen. Panels show tissue stained with either isotype control antibody (i) or βIII-tubulin antibody (ii-iv). The isotype control was negative and tumor elements had strong immunoreactivity for βIII-tubulin. Panel iv demonstrates an absence of βIII-tubulin staining in normal acinar cells (region marked by dashed border) and normal ductal cells (arrow) away from the tumor. B) Western blot analysis for βI-, βII-and βIII-tubulin in protein extracts from pancreatic cancer cell lines (MIA Paca-2, Panc-1, HPAF-II) versus normal human non-tumorigenic pancreatic ductal epithelial cells (HPDE). GAPDH was used as a loading control. C) Densitometry analysis of βIII-tubulin expression normalized to GAPDH expression demonstrates that βIII-tubulin is significantly increased in all 3 pancreatic cancer cell lines compared to HPDE cells (*p<0.05; n=3).

βIII-tubulin was expressed at high levels in pancreatic tumor cells, while absent in the acinar and normal ductal cells in PDA tissue (Figure 1A). To determine whether the expression pattern was specific to βIII-tubulin, we also examined the levels of another β-tubulin isotype, βII-tubulin, which has been shown to be differentially expressed in tumor cells [27-29]. It too was present at high levels in pancreatic tumor cells, however in contrast to βIII-tubulin, it was also present in acinar and normal ductal cells (Supplementary Figure 1). Next, we measured βIII-tubulin expression by western blotting in cell lysates from 3 different pancreatic cancer cell lines derived from primary (MiaPaCa-2, Panc-1) and metastatic (HPAF-II) sites. βIII-tubulin levels were significantly higher in all 3 pancreatic cancer cell lines compared to normal non-tumorigenic human pancreatic ductal epithelial (HPDE) cells (Figure 1B). βII-tubulin was also higher in 2/3 pancreatic cancer cell lines (MiaPaCa-2 and Panc-1) compared to HPDE cells (Figure 1B). Notably, βI-tubulin, which is constitutively expressed in most tissues, was expressed at similar levels in the pancreatic cancer cell lines and the normal HPDE cells (Figure 1B).

### Potent and specific knockdown of βIII-tubulin in pancreatic cancer cells

**Figure 2:**
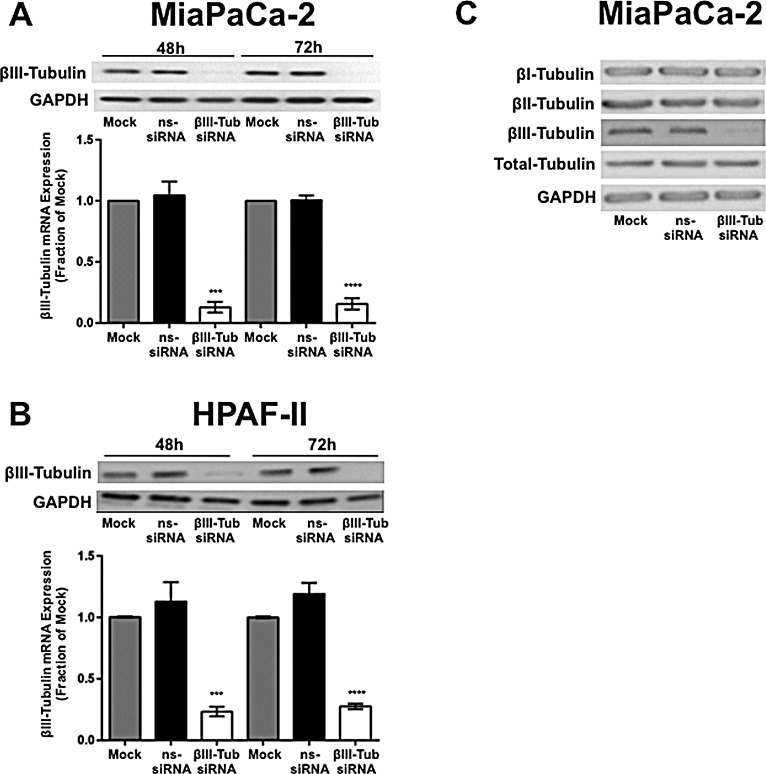
βIII-tubulin silencing in pancreatic cancer cell lines A) Top panel, Western blot analysis of βIII-tubulin silencing in protein extracts from MiaPaCa-2 cells. Cell lysates were harvested from cells 48h or 72h after transfection with mock, control siRNA (ns-siRNA), or βIII-tubulin siRNA (βIII-Tub siRNA). GAPDH was used as a loading control. Bottom graph, real-time PCR analysis of βIII-tubulin silencing in MiaPaCa-2 cells. RNA was harvested from cells 48h or 72h after transfection with mock, ns-siRNA, or βIII-tub siRNA. βIII-tubulin mRNA levels were normalized to 18S mRNA. B) as per A, except cell extracts were obtained from HPAF-II cells. Asterisks indicate significance (** p≤0.01, ** p≤0.01; n=3). C) Representative Western blots for βI-, βII-, βIII-tubulin and total tubulin in protein extracts from MiaPaCa-2 cells transfected with mock, ns-siRNA, or βIII-Tub siRNA (n=3). GAPDH was used as a loading control.

To examine whether βIII-tubulin could be suppressed in pancreatic cancer cells, we transfected two-independent pancreatic cancer cell lines (MiaPaCa-2 and HPAF-II) with βIII-tubulin siRNA. 48h and 72h post transfection, βIII-tubulin expression was measured. Knockdown of βIII-tubulin was observed at the gene level in both cell lines (MiaPaCa-2, 84.4 ± 2.6% knock-down; HPAF-II, 76.8 ± 1.1% knock-down relative to control-siRNA; 72h post-transfection) (Figure 2A and B). This correlated to knockdown (>90%) of βIII-tubulin at the protein level (Figure 2A and B). Knockdown of βII-tubulin was also observed when pancreatic cancer cells (MiaPaCa-2 and HPAF-II) were treated with βII-tubulin siRNA (Supplementary Figure 2).

To confirm that knockdown of βIII-tubulin was specific and did not cause compensational changes in the expression of other major β-tubulin isotypes, MiaPaCa-2 cells were transfected with βIII-tubulin siRNA and 72h later the levels of total β-tubulin, βI-tubulin and βII-tubulin were measured. No change in the expression of the above β-tubulins were observed following βIII-tubulin silencing (Figure 2C).

### βIII-tubulin silencing decreases clonogenicity and increases sensitivity to chemotherapeutic drugs in pancreatic cancer cells

**Figure 3:**
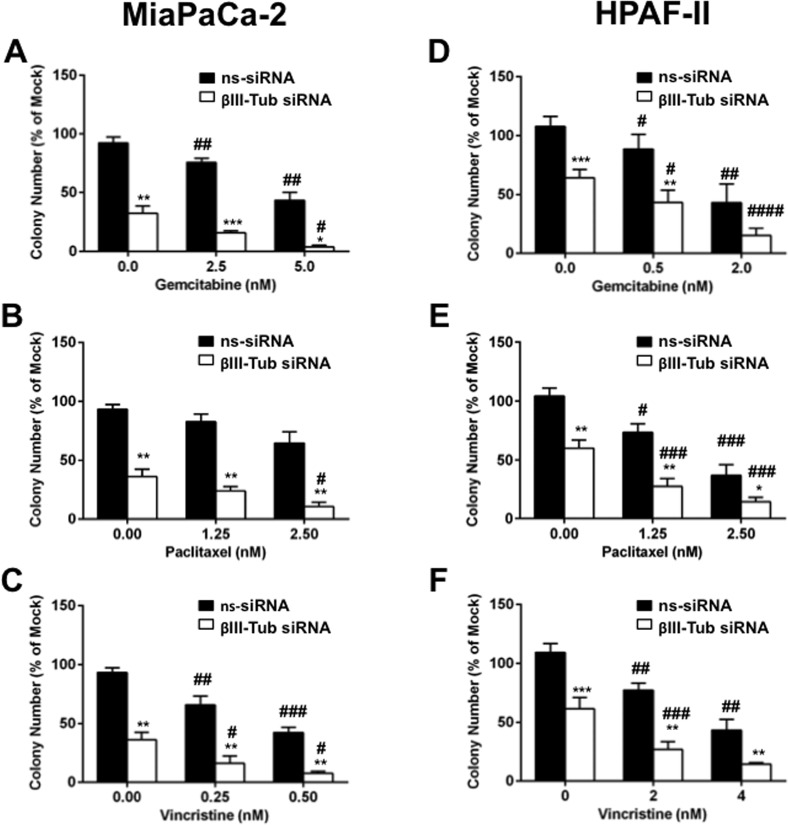
The effect of βIII-tubulin silencing on pancreatic cancer cell clonogenic capacity A-C) Bars represent the number of MiaPaCa-2 colonies (mean±s.e.m. as a % of mock) that formed from low density seeding following transfection with mock, control siRNA (ns-siRNA), or βIII-tubulin siRNA (βIII-Tub siRNA) and 72h culture in titrations of Gemcitabine (A), Paclitaxel (B) or Vincristine (C). D-F) as per A-C, except experiments were carried out with HPAF-II cells. Asterisks indicate significance relative to ns-siRNA of the same drug dose (*** p≤0.001, **** p≤0.0001; n=5). Hashes indicate significance relative to 0 nM drug concentration of the same siRNA (# p≤0.05, ## p≤0.01, #### p≤0.0001; n=5).

To establish the functional role of βIII-tubulin in pancreatic cancer, we determined the effect of silencing βIII-tubulin expression on the clonogenic potential of pancreatic cancer cells in the absence or presence of chemotherapeutic drugs. Silencing βIII-tubulin in the absence of chemotherapy significantly reduced the ability of pancreatic cancer cells (MiaPaCa-2 and HPAF-II) to form colonies compared to controls (Figures 3A and 3D). Moreover, the number of pancreatic cancer cell colonies was further decreased in the presence of the chemotherapy agents paclitaxel, vincristine, and gemcitabine (used in the first-line treatment of pancreatic cancer) when compared to controls (ns-siRNA) (Figures 3A-3F). In contrast, silencing βII-tubulin had no effect on pancreatic cancer cell colony formation in the absence or presence of chemotherapy drugs (Supplementary Figure 3A-3F). Silencing βIII-tubulin or βII-tubulin had no effect on cell viability or growth of normal non-tumorigenic HPDE cells (Supplementary Figure 4A and B). This suggests that βIII-tubulin may have a specific functional role in pancreatic cancer cells.

**Figure 4:**
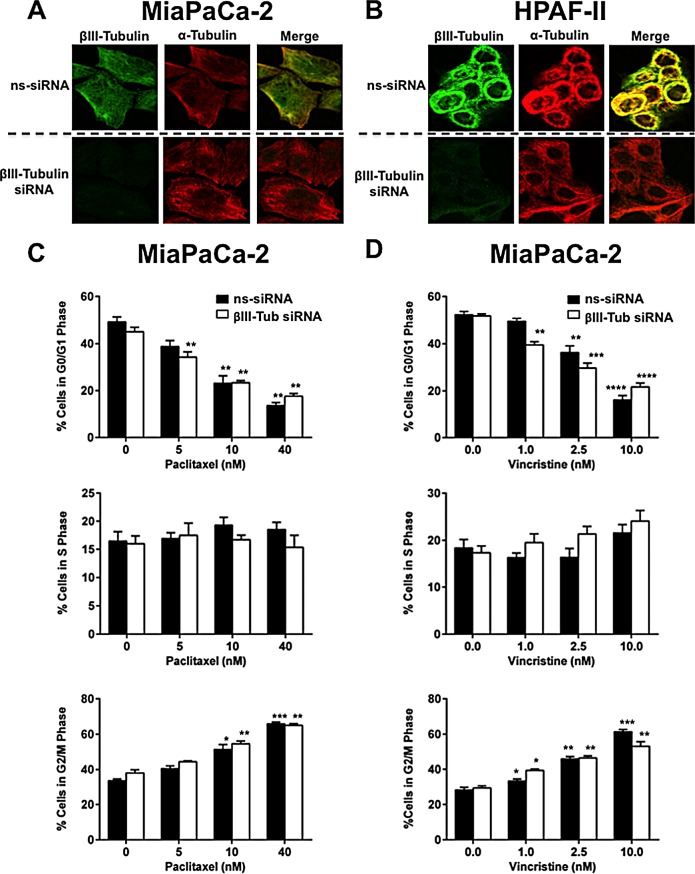
The effect of βIII-tubulin silencing on pancreatic cancer cell morphology and cell cycle A) Confocal microscopy for α-tubulin and βIII-tubulin in MiaPaCa-2 cells transfected with control siRNA (ns-siRNA) (top panels) or βIII-tubulin siRNA (βIII-Tub siRNA; bottom panels). Overlaid fluorescence images are shown in the far right panel of each row. B) as per A, except HPAF-II cells were used. C-D) Cell cycle distribution was analyzed by propidium iodide staining and flow cytometry. Bars represent % of MiaPaCa-2 cells in G0/G1-phase, S-phase, or G2/M-phase (mean±s.e.m.). 72h post-transfection with either ns-siRNA or βIII-Tub siRNA cells were incubated for eight hours with Paclitaxel (C) or Vincristine (D). Asterisks indicate significance relative to the no drug control of the same siRNA (* p≤0.05, *** p≤0.001, **** p≤0.0001; n=5).

To determine whether silencing βIII-tubulin would affect the structure of the microtubule cytoskeleton of pancreatic cancer cells, MiaPaCa-2 and HPAF-II cells were transfected with βIII-tubulin- or control siRNA, and stained with fluorescent antibodies against total α-tubulin (red) to visualize the microtubule cytoskeleton and βIII-tubulin (green). Suppression of βIII-tubulin, did not affect the structural integrity of the microtubule cytoskeleton (Figure 4A and 4B).

Next, we examined whether the increased sensitivity of pancreatic cancer cells to TBAs (paclitaxel and vincristine) following silencing of βIII-tubulin was due to increased disruption of mitosis. MiaPaCa-2 and HPAF-II cells were transfected with βIII-tubulin siRNA and then treated with increasing concentrations of paclitaxel (microtubule-stabilizing agent) or vincristine (microtubule-destabilizing agent) and their cell cycle measured. We observed increased accumulation of both control (ns-siRNA) and βIII-tubulin-siRNA treated cells in G2/M phase, along with a corresponding decrease in G0/G1 phase with increasing concentrations of paclitaxel (Figure 4C) and vincristine (Figure 4D). However, βIII-tubulin knockdown did not markedly affect cell cycle distribution, relative to controls (ns-siRNA; Figure 4C-D), suggesting that the decreased clonogenic potential of βIII-tubulin knockdown cells in the absence or presence of TBAs was not due to enhanced disruption of the cell cycle.

### βIII-tubulin silencing induces apoptosis in pancreatic cancer cells

**Figure 5:**
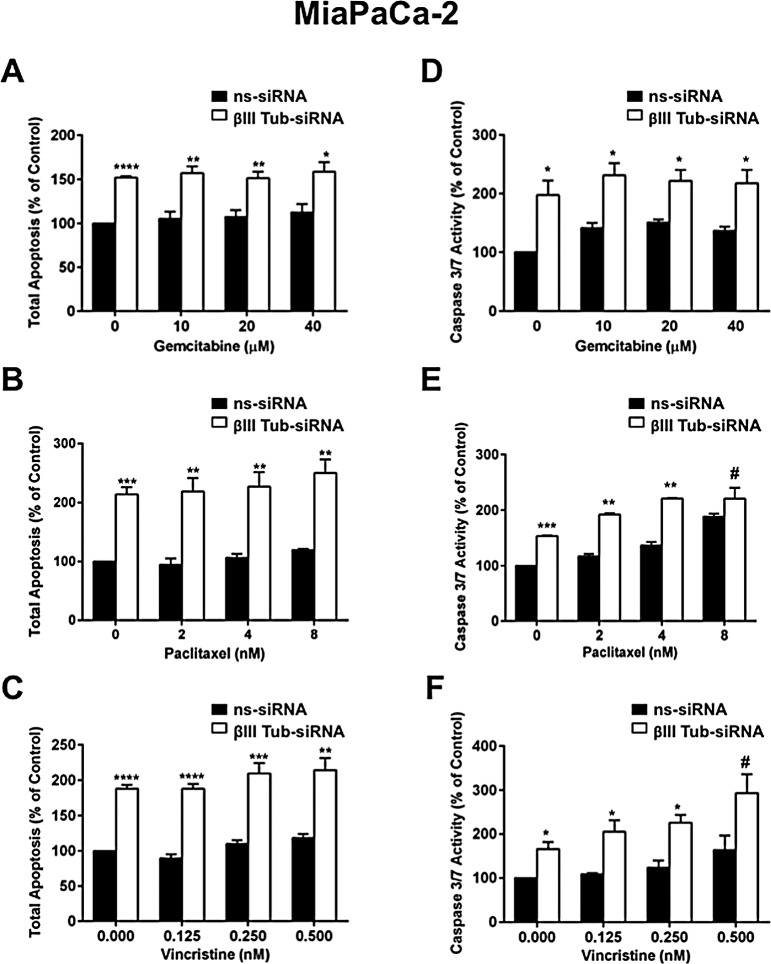
βIII-tubulin silencing induces apoptosis in MiaPaCa-2 cells A-C) MiaPaCa-2 cells transfected with control siRNA (ns-siRNA) or βIII-tubulin siRNA (βIII-Tub siRNA). 48h post-transfection cells were cultured in titrations of Gemcitabine (A), Paclitaxel (B), or Vincristine (C) for 24h. Bars represent the fraction of total MiaPaCa-2 cells that are apoptotic (mean+s.e.m. as a % of ns-siRNA no drug control) as determined by Annexin V and 7AAD staining. Asterisks indicate significance relative to ns-siRNA controls of the same drug dose (* p≤0.05, ** p≤0.01, *** p≤0.001; n=3-4). D-F) MiaPaCa-2 cells transfected with ns-siRNA or βIII-Tub siRNA were cultured in titrations of Gemcitabine (D), Paclitaxel (E), or Vincristine (F) for 24h. Bars represent caspase 3/7 activity (mean±s.e.m. as a % of ns-siRNA no drug control). Asterisks indicate significance relative to ns-siRNA controls of the same drug dose (* p≤0.05, ** p≤0.01, *** p≤0.001; n=4-5).

To investigate whether apoptosis was responsible for the reduction in the number of pancreatic cancer cell colonies, MiaPaCa-2 cells were transfected with βIII-tubulin or control siRNA (ns-siRNA) and then treated with or without chemotherapy drugs. βIII-tubulin suppression in pancreatic cancer cells induced a marked increase in apoptosis (Annexin V and 7AAD) in the absence of chemotherapy drugs (Figure 5A-F). This was echoed by a significant increase in caspase 3/7 activity, in βIII-tubulin knockdown cells (Figure 5D-F). Furthermore, the increase in apoptosis was sustained in the presence of chemotherapy drugs (Figure 5D-F). These data suggest that the decreased clonogenic potential in pancreatic cancer cells is mediated via increased apoptosis. To the best of our knowledge this is the first report to demonstrate a role for βIII-tubulin in regulating cell survival in tumor cells in the absence of chemical and /or environmental stress.

### βIII-tubulin silencing reduces the tumorigenic potential of pancreatic cancer cells

**Figure 6:**
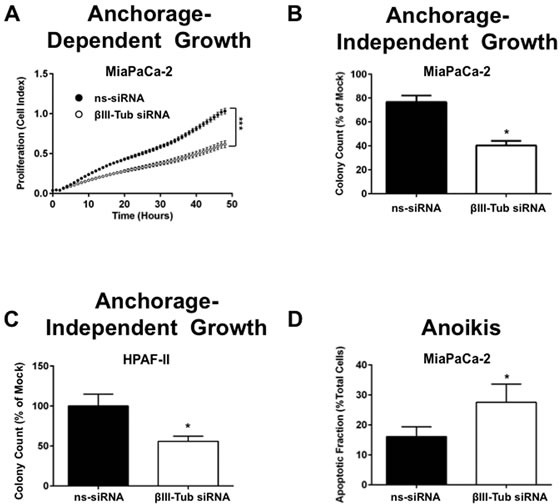
βIII-tubulin silencing reduces both anchorage dependent and independent pancreatic cancer cell growth A) xCelligence assay of MiaPaCa-2 cells transfected with ns-siRNA or βIII-tubulin siRNA (βIII-Tub siRNA). 72h post-transfection cells were seeded into xCelligence plates. Circles represent cell index (mean±s.e.m.) at hourly time points, and are directly related to cell number. Asterisks indicate significance (*** p≤0.001; n=3). B) MiaPaCa-2 cells transfected with mock, ns-siRNA or βIII-Tubulin siRNA were embedded in soft-agarose at 48h post-transfection and allowed to form colonies. Bars represent the number of colonies that formed (mean+s.e.m. as a % of mock). Asterisks indicate significance (*p≤0.05; n=3). C) As per B, except experiments were carried out with HPAF-II cells (* p≤0.05; n=3). D) MiaPaCa-2 cells were transfected with ns-siRNA or βIII-tubulin siRNA and 24h post-transfection cells were cultured under anchorage independent conditions for a further 48h (wells coated with Poly-HEMA). Bars represent the apoptotic fraction determined by Annexin V and 7AAD staining (mean±s.e.m.). Asterisks indicate significance (*** p≤0.001; n=3).

Our drug-clonogenic and apoptosis results suggested that βIII-tubulin might be playing a survival role in pancreatic cancer cells. Therefore, we investigated the effect of βIII-tubulin suppression on pancreatic cancer cell anchorage-dependent and -independent growth, as well as anoikis (anchorage-independent programmed cell death). Tumor cells with high tumorigenic and metastatic potential have acquired mechanisms to grow and survive under both anchorage-dependent and independent conditions, and develop mechanisms to resist anoikis. First, anchorage-dependent cell growth was measured in real-time using the xCELLigence platform. Consistent with our drug-clonogenic results, we observed significantly reduced cell proliferation in MiaPaCa-2 cells transfected with βIII-tubulin siRNA relative to controls (ns-siRNA) over 48h (Figure 6A). Next, we assessed the effects of βIII-tubulin knockdown on pancreatic cancer cell anchorage-independent growth. MiaPaCa-2 and HPAF-II cells were transfected with βIII-tubulin siRNA or control siRNA (ns-siRNA) and then grown in soft-agar for 14 days. Knockdown of βIII-tubulin significantly reduced the number of cell colonies formed (Figure 6B and 6C). Finally, to investigate whether the reduction in cell colonies was in part due to anoikis, MiaPaCa-2 cells transfected with control or βIII-tubulin siRNA were cultured in suspension for 48h. We observed a significant increase in the apoptotic fraction of MiaPaCa-2 cells with knockdown of βIII-tubulin (Figure 6D). Collectively, these results provide strong evidence that βIII-tubulin plays an important role in promoting the growth and metastatic potential of pancreatic cancer cells.

### Stable suppression of βIII-tubulin reduces tumor growth and metastases in an orthotopic murine model of pancreatic cancer

**Figure 7:**
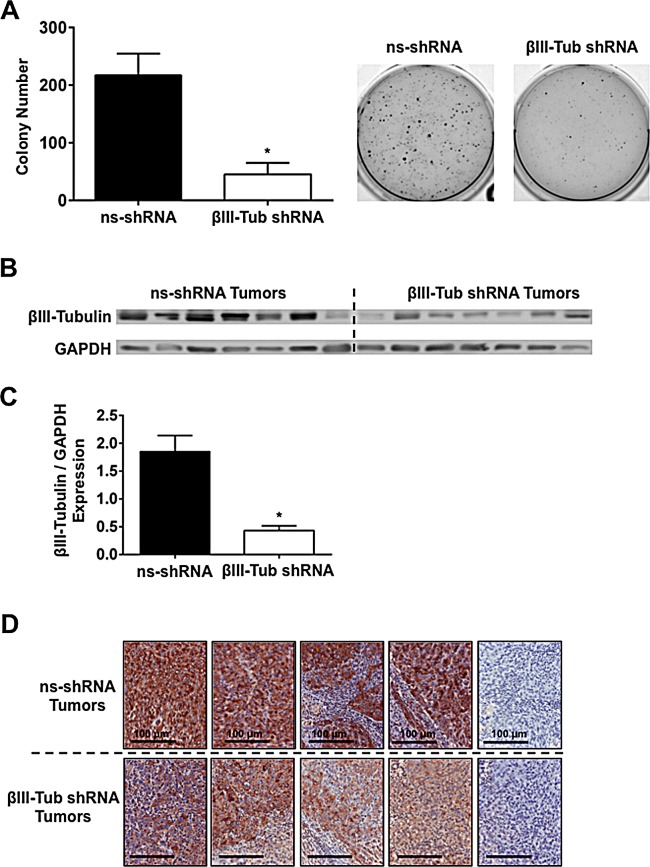
βIII-tubulin shRNA expressing MiaPaCa-2 cells have reduced anchorage-independent growth and potent long-term βIII-tubulin knockdown *in vivo* A) MiaPaCa-2 cells stably expressing βIII-Tubulin (βIII-Tub) or control shRNA (ns-shRNA) were embedded in soft-agarose and allowed to form colonies. Bars represent the number of colonies that formed (* p≤0.05; n=3). Representative micrographs showing colony formation in MiaPaca-2 cells stably expressing control (ns-shRNA) or βIII-Tubulin (βIII-Tub) shRNA. B) Western blot for βIII-tubulin in protein extracts from non-silencing shRNA (ns-shRNA) and βIII-Tubulin shRNA (βIII-Tub shRNA) tumors after eight weeks of *in vivo* growth. GAPDH was used as a loading control. C) Densitometry analysis of βIII-tubulin expression normalized to GAPDH expression demonstrates that βIII-tubulin is significantly decreased in pancreatic tumors stably expressing βIII-tubulin shRNA compared to control (ns-shRNA) expressing tumors (*p<0.05; n=7). D) Immunohistochemistry for βIII-tubulin in tissue sections from primary tumors expressing (i-v) ns-shRNA or (vi-ix) βIII-Tubulin shRNA.

**Figure 8:**
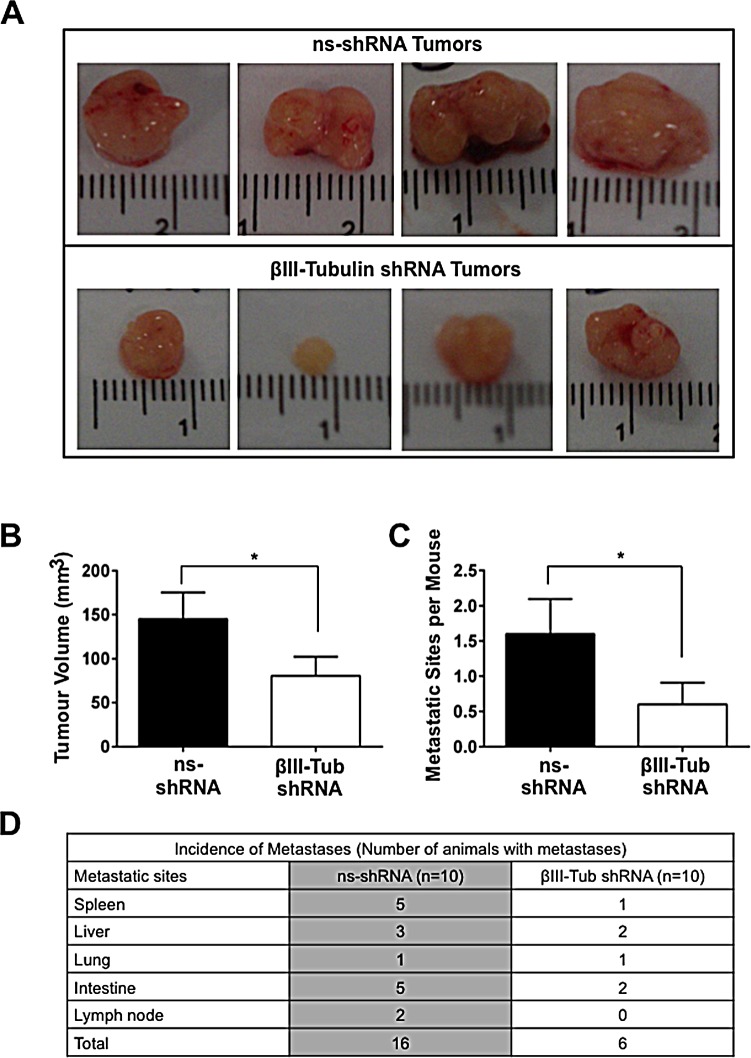
The effect of βIII-tubulin silencing on orthotopic tumor growth and metastases A) Representative photomicrographs of primary tumors expressing control (ns-shRNA) or βIII-tubulin (βIII-Tub) shRNA eight weeks post-implantation (n=4 individual mice per group). B) Bars represent tumor volume (mean±s.e.m.) of primary pancreatic tumors eight weeks post-implantation. Asterisks indicate significance (* p≤0.05; n=9 for ns-shRNA and n=10 for βIII-tubulin shRNA). C) Bars represent the number of metastatic sites per mouse (mean±s.e.m.) eight weeks post-implantation. Asterisks indicate significance (* p≤0.05). D) Table showing the total number of metastases detected in each organ for ns-shRNA and βIII-Tub shRNA tumors.

To extend our findings *in vivo*, we generated MiaPaCa-2 cells that stably expressed luciferase and a βIII-tubulin shRNA construct. Cells stably expressing the same construct but with a non-functional shRNA (ns-shRNA) served as controls. These cells possessed potent knockdown of βIII-tubulin compared to control shRNA cells (Supplementary Figure 5A and 5B). Notably, cells with stable suppression of βIII-tubulin had a significant decrease in anchorage-independent growth when compared to controls (ns-shRNA) (Figure 7A). These results concurred with our data using transient knockdown of βIII-tubulin (Figure 6B). Cells were implanted into the pancreas of mice, and allowed to grow for 8 weeks before tumors along with the spleen, liver, kidneys, intestines, and heart/lungs were harvested together with any lymph nodes that exhibited signs of metastases. βIII-tubulin knockdown in the tumors (8 weeks post implantation) containing βIII-tubulin shRNA was confirmed (Figures 7B-7D). Pancreatic tumors expressing βIII-tubulin shRNA had significantly reduced tumor volume relative to control shRNA tumors (Control: 145.0 ± 30.5 mm^3^, βIII-Tubulin shRNA: 80.6 ± 21.9 mm^3^, p<0.05; Figure 8A-B). The incidence of metastases was reduced by 30% in mice with tumors expressing βIII-tubulin shRNA (4/10 mice had metastases) versus tumors expressing control shRNA (7/10 mice had metastases). Metastases that were detected by *ex vivo* imaging of individual organs on a Xenogen IVIS platform (Supplementary Figure 6A) were found to be reduced by 62.5% in the βIII-tubulin shRNA pancreatic tumors, relative to controls (Figure 8C-D). Metastases detected by *ex vivo* imaging were confirmed by histology (Supplementary Figure 6B) and immunohistochemistry (Supplementary Figure 6C).

## DISCUSSION

Pancreatic cancer is chemoresistant and metastatic, making this a lethal cancer. There is mounting evidence to show that βIII-tubulin is dysregulated in tumor cells, and its increased expression is correlated to poor survival [30-33]. However, the role of βIII-tubulin in pancreatic cancer is unknown. We report novel functions for βIII-tubulin in regulating pancreatic cancer cell growth and survival in the absence and presence of chemotherapeutic drugs. In addition, we demonstrate that silencing βIII-tubulin expression in pancreatic cancer cells results in reduced tumor growth and metastases *in vivo*.

In health, βIII-tubulin expression is restricted to neuronal tissue and testicular sertoli cells [19, 20]. However, clinical studies report high levels of βIII-tubulin in different tumors including breast, lung, ovarian, gastric and melanoma [13-18]. Recently, high βIII-tubulin expression was reported in prostate cancer, and was linked to high Gleason grade, advanced tumor stage and local metastases [31]. In this study we demonstrated that βIII-tubulin was expressed at high levels in pancreatic tumor cells, while absent in the acinar and normal pancreatic ducts in tissue specimens collected after surgical resection. We also showed increased expression of βIII-tubulin in pancreatic cancer cell lines compared to primary cultures of HPDE cells. Our results are in accordance with a study by Lee et al. [26], which reported βIII-tubulin expression in advanced PDA tissue samples. However, prior to the present study, there have been no studies to elucidate its functional role.

Using RNAi we demonstrated that silencing βIII-tubulin in pancreatic cancer cells reduced their ability to form cell colonies. Moreover, the number of colonies was further reduced when the cells were treated with different chemotherapy drugs including, the anti-metabolite gemcitabine (used in the first-line treatment of pancreatic cancer) and the TBAs paclitaxel and vincristine [albumin bound paclitaxel (Abraxane) is now used in the clinic to treat pancreatic cancer and vincristine while not a pancreatic cancer treatment, was used in this study to help delineate βIII-tubulin's function]. Knockdown of βII-tubulin, which was also overexpressed in pancreatic cancer cells, had no effect on the clonogenic potential of pancreatic cancer cells in the absence or presence of chemotherapy drugs. This result is in contrast to a previous study in NSCLC cells which showed that silencing βII-tubulin expression increased sensitivity to Vinca alkaloids (25). Therefore, it is possible that the ability of pancreatic cancer cells to form colonies in the absence or presence of chemotherapy drugs may be β-tubulin isotype specific. Moreover, βIII-tubulin appears to have a tumor cell-specific effect as silencing its expression in normal HPDE cells had no effect on their cell viability or proliferation.

A role for β-tubulin isotypes in regulating chemosensitivity in tumor cells has been described. Gan et al. [22] showed that silencing βIII-tubulin in NSCLC cells reduced their clonogenic capacity in the presence of chemotherapy. This finding was extended to an *in vivo* setting by McCarroll et al. [24], which reported that stable suppression of βIII-tubulin in NSCLC cells increased survival in mice when treated with cisplatin. More recently, βIII-tubulin was shown to be involved in regulating docetaxel sensitivity in castrate-resistant prostate cancer cells [31]. Interestingly, Gan et al. [28] reported that suppression of βII- or βIVb-tubulin in NSCLC cells increased sensitivity to only one class of chemotherapy drugs known as Vinca Alkaloids. These studies reinforce the concept that individual β-tubulin isotypes may have specific functional roles in tumor cells, with βIII-tubulin appearing to be important in regulating sensitivity to broad classes of chemotherapy drugs. However, despite the increasing number of studies that highlight the importance of βIII-tubulin in tumor cells, its mode of action has yet to be fully determined.

In an attempt to understand how βIII-tubulin is exerting its effect on pancreatic cancer cells we first examined whether silencing its expression influenced the structural integrity of the microtubule cytoskeleton, given the importance of microtubule proteins in regulating the shape and structure of cells. Silencing βIII-tubulin expression in pancreatic cancer cells had no effect on the structure of the microtubule cytoskeleton. Next, we assessed whether silencing βIII-tubulin impacted the cell cycle given the importance of microtubule proteins in regulating mitosis. No significant effect on the cell cycle was observed. We also showed that silencing βIII-tubulin did not potentiate the anti-mitotic effect of TBAs in pancreatic cancer cells. Together, these results indicate that the observed decrease in the clonogenic potential of pancreatic cancer cells with suppressed βIII-tubulin in the absence or presence of chemotherapeutic drugs does not involve modulation of the microtubule cytoskeleton or the cell cycle.

Finally, to determine whether this decrease in the clonogenic potential of pancreatic cancer cells with suppressed βIII-tubulin was due to increased sensitivity to cell death, we measured apoptosis in pancreatic cancer cells. We demonstrated that silencing βIII-tubulin induced cell death in pancreatic cancer cells which was sustained in the presence of chemotherapy. It appeared that the intrinsic apoptotic pathway was involved as evidenced by increased caspase 3/7 activity. This is the first study to report a significant induction of apoptosis in tumor cells with suppressed βIII-tubulin in the absence of cellular stress. Previously, induction of apoptosis in tumor cells with knockdown of βIII-tubulin was observed only when cells were treated with chemotherapeutic drugs [22, 24]. Therefore, it appears that pancreatic cancer cells are highly sensitive to suppression of βIII-tubulin, and that this protein may provide these cells with a survival advantage. Indeed, evidence in other cell types suggest that βIII-tubulin may be part of a cell survival pathway. For instance, its expression levels can be modulated by different types of cell stress. In two separate studies, Raspaglio et al [34, 35] demonstrated increased βIII-tubulin expression in ovarian cancer cells exposed to hypoxia or nutrient deprivation. Under these conditions βIII-tubulin was shown to bind to important signaling proteins such as pro-survival kinase PIM1 [36]. This protein kinase is involved in promoting chemoresistance, and its expression levels have been correlated with aggressive disease in pancreatic cancer [37, 38]. Therefore, it is possible that the increased levels of βIII-tubulin allow for PIM1 to exert its pro-survival effect in pancreatic cancer cells. In addition, a glycosylated and phosphorylated form of βIII-tubulin has been identified in the mitochondria of cancer cells [39]. It may be possible that βIII-tubulin is involved in modulating apoptosis via the mitochondria. Studies aimed at understanding how βIII-tubulin hypersensitizes pancreatic cancer cells to apoptosis are under investigation in our laboratory.

To determine whether suppression of βIII-tubulin would affect the tumorigenic and metastatic potential of pancreatic cancer cells, we silenced βIII-tubulin expression in pancreatic cancer cells and measured anchorage-dependent and independent-cell growth. Knockdown of βIII-tubulin resulted in a significant reduction in anchorage-dependent and independent growth. The decrease in anchorage-independent cell growth was associated with increased anoikis (anchorage-independent apoptosis), reinforcing the link between βIII-tubulin silencing and induction of apoptosis in pancreatic cancer cells. Indeed, tumor cells with high metastatic potential have developed mechanisms of resistance to this form of apoptosis [40]. To establish whether the decreased tumorigenic potential in pancreatic cancer cells with suppressed βIII-tubulin would translate *in vivo*, we generated pancreatic cancer cells which stably expressed βIII-tubulin shRNA. We showed for the first time that when these cells were implanted into the pancreas of mice there was decreased primary tumor growth and metastases. Together, these data demonstrate that βIII-tubulin is important in providing pancreatic cancer cells with a key survival advantage, thus allowing them to grow and metastasize.

Collectively, this work has identified a novel role for βIII-tubulin in promoting pancreatic cancer growth and survival. Identification of βIII-tubulin as a therapeutic target has the potential to refine personalized medicine for patients with this malignancy. The relatively limited impact of chemotherapy in this malignancy does require identification of agents with alternative anti-tumor activity. Drugs targeting this system would be predicted to have limited effects on normal tissues. Therefore they lend themselves to prolonged use, such as in the adjuvant setting. In particular the anti-metastatic effect shown *in vivo* encourages exploration of their use in the adjuvant setting and in conjunction with or sequential to chemotherapy.

## MATERIALS AND METHODS

### Cell culture

Human pancreatic cancer cells (MiaPaCa-2, Panc-1 and HPAF-II) were obtained from ATCC and cultured as described [41]. Normal Human Pancreatic Ductal Epithelial (HPDE) cells (a kind gift from Ming Tsao, Ontario Cancer Institute) were grown in Keratinocyte-serum-free (KSF) medium supplemented with 50 mg/ml bovine pituitary extract (BPE) and 5 ng/ml epidermal growth factor (EGF) as described [42].

### Immunohistochemistry

Human PDA tissue specimens were collected by surgical removal. The use of these sections was approved by UNSW Human Research Ethics Committee (HCEC# HC14039). Immunohistochemistry was performed on paraffin-embedded human or mouse tumor tissue sections as described [24, 41, 43]. Antibodies used were, anti-βIII-tubulin (1:200) (Chemicon), anti-βII-tubulin (1:200) (Covance), anti-luciferase (1:50) (Biovision incorporated).

### siRNA transfection

Pancreatic cancer cells and HPDE cells were transfected with siRNAs using Lipofectamine 2000 (Invitrogen). All cells were transfected with smart pool On-Target Plus siRNAs designed against βII-Tubulin (Thermoscientific, Cat. L-008260-00), βIII-Tubulin (Thermoscientific, Cat. L-020099-00), or non-silencing control (Thermoscientific, Cat. D-001810-10-20).

### Real time quantitative PCR (qPCR)

Total RNA was extracted from pancreatic cancer cells and HPDEs, and transcribed to cDNA as described [24, 43]. qPCR was performed using the QuantiFast SYBR Green PCR kit (Qiagen) as described [24, 43]. Primer sequences were: βIII-Tubulin forward primer, 5′-GCGAGATGTACGAAGACGAC-3′; βIII-Tubulin reverse primer, 5′-TTTAGACACTGCTGGCTTCG-3′; βII-Tubulin forward primer, 5′-AAAGAATTCGACGCCACGGCCGACGAA CAAGGG-3′; βII-Tubulin reverse primer, 5′-AAAAGCTTACAAACGTTTATGTGATTTTAG-3′. All data were normalized to the 18S gene (Quantitect Primer Assay, Qiagen).

### Western blot analysis

Western blot analysis was performed using the following antibodies: anti-βIII-tubulin (Chemicon), anti-βII-tubulin (Covance), anti-βI-tubulin (Covance) and anti-GAPDH (Abcam) as described [22, 24, 28]. The blots were scanned using LAS4000 scanner and quantified using ImageQuant TL (GE Healthcare).

### Immunofluorescence staining

24h post-transfection with siRNA, cells were seeded onto glass chamber slides and allowed to adhere for 48h. The slides were fixed in 4% paraformaldehyde and immunofluorescence staining was performed as described [22, 43, 44]. Primary and secondary antibodies used were, anti-βIII-tubulin (1:500), anti-α-tubulin (1:500), anti-goat AlexaFluor-488 or AlexaFluor-555 (1:1000). Images were captured using a Leica confocal microscope.

### Cell proliferation assays

HPDE cell proliferation was measured 72h post-transfection using the Cell Counting Kit-8 (CCK-8) kit (Dojindo) as described [24, 41]. MiaPaCa-2 proliferation was measured on an xCELLigence platform (ACEA Biosciences). Cells were seeded 72h post-transfection at 3000 cells/well into 96-well E-Plates (ACEA Biosciences), then transferred into an xCELLigence in a humidified 37^o^C chamber in 5% CO_2_. Cell Index (Proliferation) was measured hourly for 48h.

### Cytotoxic drug-clonogenic assays

Following siRNA transfection cytotoxic drug-clonogenic assays were performed as described [22, 24, 28].

### Cell cycle analysis

72h post siRNA transfection, MiaPaCa-2 cells were incubated in Taxol (0-40 nM) or Vincristine (0-10 nM) for 8h, and cell cycle measured as described [44].

### Detection of apoptosis

48h after siRNA transfection, pancreatic cancer cells were treated with culture medium containing cytotoxic drugs for 24h. Cell death was measured using the Annexin V-PE-7-AAD-FITC reagent (Millipore) as described [41, 45, 46]. Caspase 3/7 activity was analyzed using a Caspase-Glo luminescent based assay as previously described [24, 41]. Briefly, pancreatic cancer cells were treated with siRNA as above and seeded into a 96-well white opaque plate and a corresponding clear 96-well plate. 24h post-cytotoxic drug treatment, cells in the white opaque plate were incubated with caspase 3/7 reagent for 2h at room temperature and luminescence measured with a luminometer (PerkinElmer Victor 3). Cells in the clear 96-well plate were incubated with CCK-8 reagent for 1h at room temperature and cell viability was determined by measuring absorbance at 450nm. Caspase 3/7 activity was then normalized to these values.

### Soft-agar assay

MiaPaCa-2 and HPAF-II cells were seeded in 0.33% agar in X2 growth medium on a 5% agar layer in 6 well plates, 48h post-siRNA transfection. Colonies were allowed to grow over 3 weeks, after which plates were stained with MTT and visualized on an ImageQuant LAS4000 luminometer (GE Healthcare). Colonies were counted as described [24].

### Anoikis assay

1 ml of 12 mg/ml poly 2-hyroxyethyl methacrylate (Poly-HEMA) in 95% ethanol was added into each well of a 6-well culture plate and left to dry overnight. 24h after siRNA transfection MiaPaCa-2 cells were seeded into the Poly-HEMA-coated wells. Cell death was measured 48h later by annexin V-7AAD staining.

### Generation of βIII-tubulin stable short hairpin RNA (shRNA)–luciferase expressing cells

MiaPaCa-2 cells were first transfected with 2 μg of pGL4.50 (Mammalian Luciferase, Promega) using lipofectamine 2000. Clones with stable integration of the construct were isolated. A stable luciferase-expressing line was transfected with 2 μg of pGFP-V-RS empty vector (negative control), pGFP-V-RS vector containing βIII-tubulin shRNA (5′-CAGCAGATGTTCGATGCCAAGAACATGAT-3′) or pGFP-V-RS vector containing a non-effective shRNA (5′-GCACTACCAGAGCTAACTCAGATAGTACT-3′) (OriGENE) using lipofectamine 2000. Three days after transfection, the integration of the shRNA plasmid was verified by measuring βIII-tubulin expression. GFP^hi^ cells were sorted on a BD Influx Cell Sorter and propagated for 2 weeks in 1 μg/ml puromycin.

### Orthotopic pancreatic cancer mouse model

6-8 week old BALB/c nude mice were used. All animal experiments were approved by the Animal Ethics committee, UNSW (ACEC 12/7B). MiaPaCa-2 cells (1×10^6^) were implanted into the tail of the pancreas of mice as described [41, 46]. After 8 weeks, mice were sacrificed and pancreatic tumors, spleen, liver, kidneys, Intestines, heart and lungs and any enlarged lymph nodes/other organs with signs of metastases were collected. Primary tumor size was measured using microcallipers as described [24, 43]. Metastases were confirmed by macroscopic observation, post-harvest individual organ *ex-vivo* bioluminescence on the Xenogen IVIS Lumina, as described [47].

### Statistical Analyses

Data are expressed as mean ± standard error of the mean (SEM) and analyzed where appropriate using ANOVA followed by parametric Dunnett, or Student's *t* test followed by the nonparametric Wilcoxin test using the GraphPad Prism program. *P*<0.05 was considered statistically significant.

## SUPPLEMENTARY MATERIAL, FIGURES


