# Supplementary material for: βIII-Tubulin: A novel mediator of chemoresistance and metastases in pancreatic cancer
Source: Oncotarget. 2014 Dec 10;6(4):2235–49. doi: 10.18632/oncotarget.2946 (PMC4385848; doi:10.18632/oncotarget.2946)
Supplement: Supplementary file 1 [file oncotarget-06-2235-s001.pdf]

βIII-Tubulin: A novel mediator of chemoresistance and metastases in pancreatic cancer

Supplementary Material

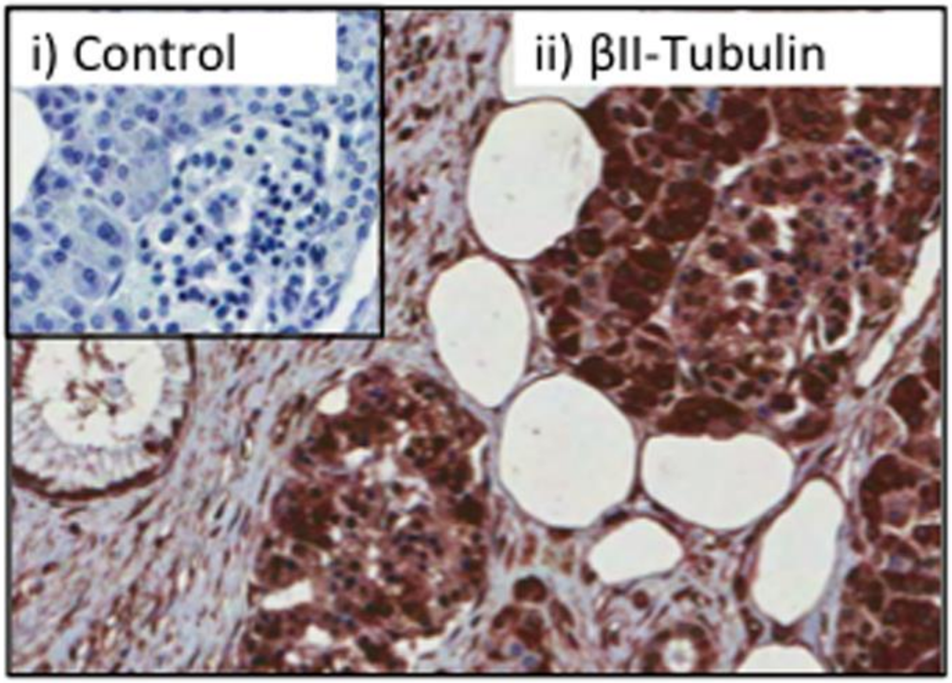

SUPPLEMENTARY FIGURE 1: βII-Tubulin is upregulated in PDA patient tissue and PDA cell lines. **A,** Immunohistochemistry for βII-Tubulin in a human PDA tissue specimen; Panels show tissue stained with either isotype control antibody (i) or βIII-Tubulin antibody (ii).

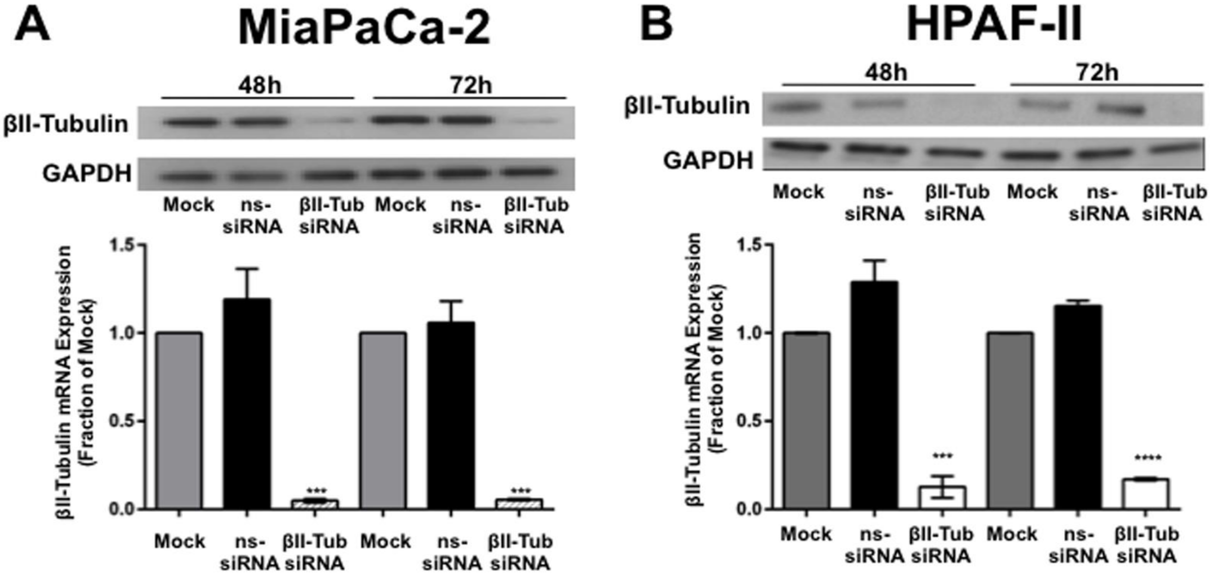

SUPPLEMENTARY FIGURE 2: βII-Tubulin silencing in PDA cell lines. **A,** top, Western blot analysis of βII-Tubulin silencing in protein extracts from MiaPaCa-2 cells. Proteins were harvested from cells 48h or 72h after transfection with vector only control (mock), non-silencing siRNA (ns-siRNA), or βIII-Tubulin siRNA (βIII-Tub siRNA). GAPDH was used as a loading control; bottom, RT-qPCR analysis of βII-Tubulin silencing in MiaPaCa-2 cells. RNA was harvested from cells 48h or 72h after transfection with mock, ns-siRNA, or βIII-Tub siRNA. βIII-Tubulin mRNA levels were normalized to 18S mRNA. **B,** as per **A,** except extracts were obtained from HPAF-II cells. Asterisks indicate significance according to student two-tailed t-test, (***, P≤0.001; ****, P≤0.0001).

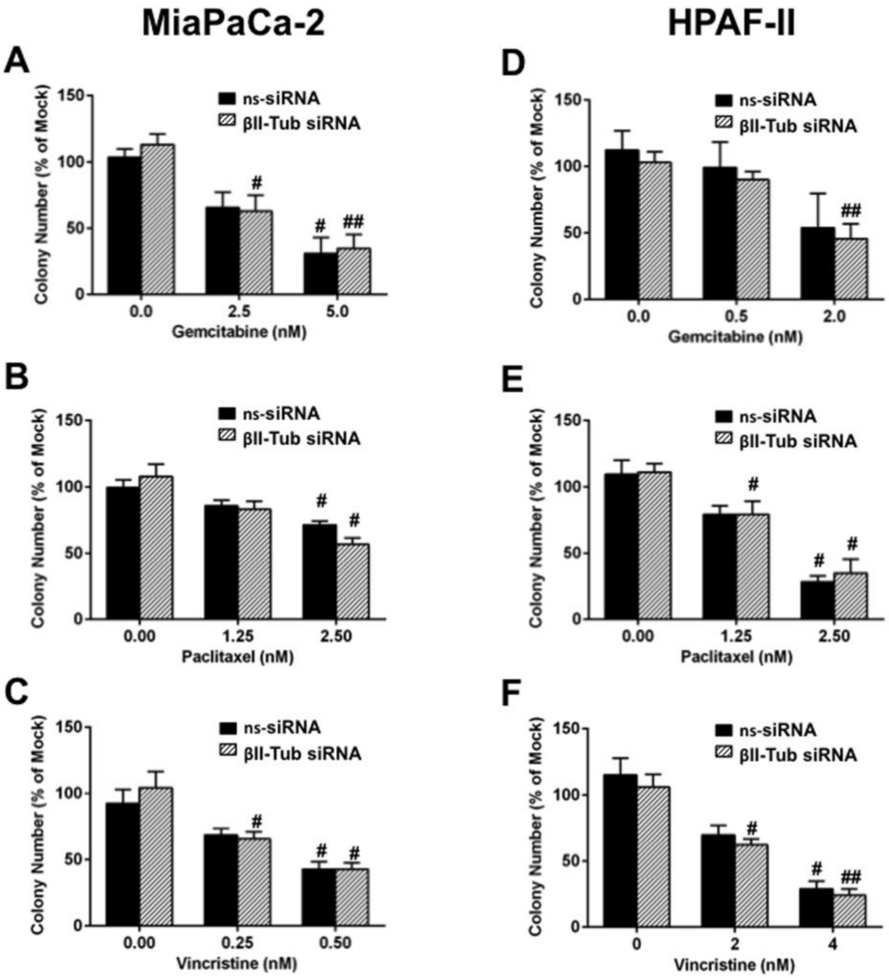

SUPPLEMENTARY FIGURE 3: The effect of βII-Tubulin silencing on PDA cell clonogenic capacity. **A-C,** Bars represent the number of MiaPaCa-2 colonies (mean+s.e.m. as a % of mock) that formed from low density seeding following transfection with mock, ns-siRNA, or βII-Tubulin siRNA (βII-Tub siRNA) and 72h culture in titrations of **A,** Gemcitabine, **B,** Taxol or **C,** Vincristine. **D-F,** As per **A-C,** except experiments were done with HPAF-II cells. Hashes indicate significance according to 1-way ANOVA, relative to 0 nM drug concentration of the same siRNA (#, P≤0.05; ##, P≤0.01; ####, P≤0.0001).

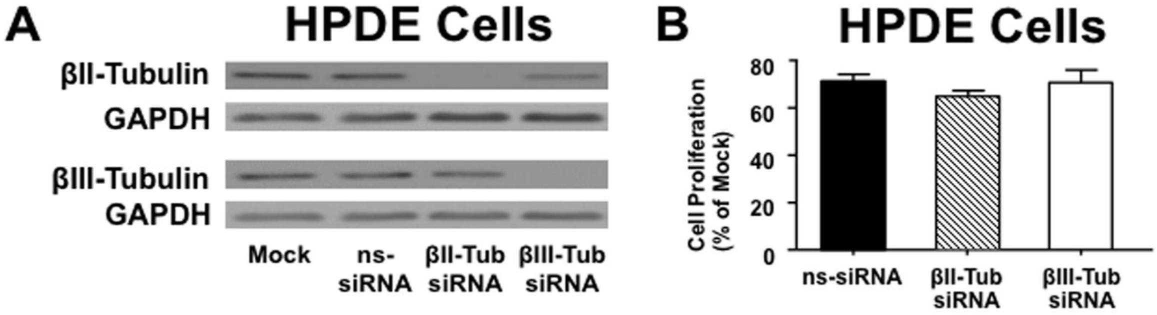

SUPPLEMENTARY FIGURE 4: The effect of silencing βII- and βIII-tubulin on normal human pancreatic ductal epithelial cell viability. **A)** Western blot analysis for βII-and βIII-Tubulin in protein extracts from normal human non-tumorigenic pancreatic ductal epithelial (HPDE) cells transfected with mock, ns-siRNA, βII-Tubulin siRNA (βII-Tub siRNA) or βIII-Tub siRNA. GAPDH was used as a loading control. **B)** Bars represent the number of HPDE cells (mean±s.e.m. as a % of mock) as determined by cell counting kit-8 proliferation assay 72h post-transfection. HPDE cells were transfected with mock, ns-siRNA, βII-Tub siRNA, or βIII-Tub siRNA (n=3).

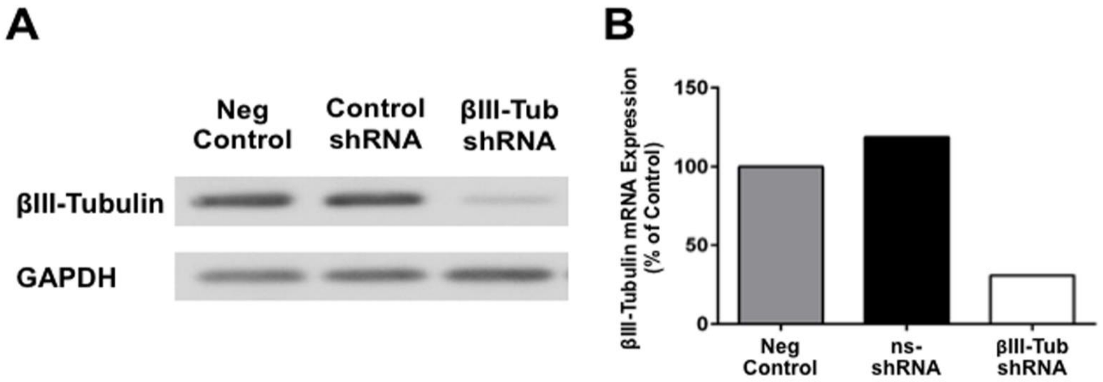

SUPPLEMENTARY FIGURE 5: βIII-Tubulin silencing in PDA cell lines. **A,** Western blot analysis for βIII-Tubulin in protein extracts from MiaPaCa-2 cells stably transfected with ns-shRNA or βIII-Tubulin shRNA (βIII-Tub shRNA). GAPDH was used as a loading control. **B,** RT-qPCR analysis of βIII-Tubulin silencing in MiaPaCa-2 stably transfected with ns-shRNA or βIII-Tub shRNA. βIII-Tubulin mRNA levels were normalized to 18S mRNA.

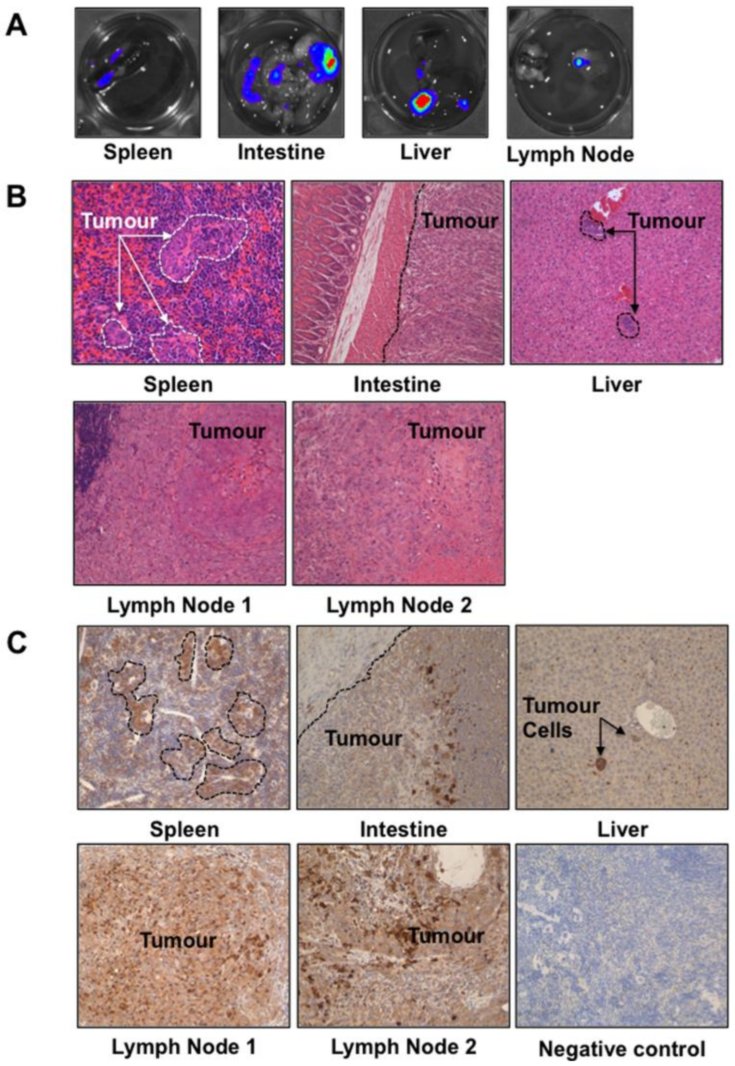

SUPPLEMENTARY FIGURE 6: Confirmation of metastases by luminescence and Immunohistochemical staining. **A,** Example of *Ex vivo* luminescence images of organs from control mice showing the presence of luminescent pancreatic tumor cells in the spleen, intestine, liver and lymph node. **B,** H&E staining of organs from control and βIII-tubulin knock-down mice that tested positive for metastases by luminescence. Both lymph nodes were superficial cervical nodes and had no normal tissue left. **C,** Immunohistochemical staining of luciferase confirming the presence of pancreatic cancer cells in the spleen, intestine, liver and lymph nodes of control mice.
